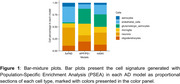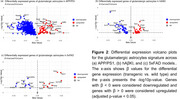# Transcriptomic profiling of glutamatergic gliotransmission‐specialized astrocytes in Alzheimer's disease mouse models

**DOI:** 10.1002/alz70855_101393

**Published:** 2025-12-23

**Authors:** Ramon Bertoldi de Souza, Christian Limberger, Gabriel Colissi Martins, Gabriel Lermen Hoffmeister, Mariana Radaelli Schmaedek, Roberta dos Santos de Oliveira, Gabriela Mantovani Baldasso, Marco Antônio De Bastiani, Eduardo R. Zimmer

**Affiliations:** ^1^ Universidade Federal do Rio Grande do Sul, Porto Alegre, Rio Grande do Sul, Brazil; ^2^ University of Cologne, Cologne, Germany; ^3^ Universidade Federal do Rio Grande do Sul, Porto Alegre, RS, Brazil; ^4^ McGill University, Montreal, QC, Canada; ^5^ Brain Institute of Rio Grande do Sul ‐ Pontifícia Universidade Católica do Rio Grande do Sul, Porto Alegre, Rio Grande do Sul, Brazil

## Abstract

**Background:**

Astrocytes, the most abundant glial cells in the brain, exhibit remarkable heterogeneity and play critical roles in maintaining brain homeostasis. Their responses to Alzheimer's disease (AD) pathology are diverse, particularly in the context of glutamate excitotoxicity — a hallmark of AD characterized by impaired astrocytic glutamate uptake. Recently, de Ceglia et al. (2023) identified a astrocyte subtype involved in glutamatergic gliotransmission, defined by the expression of genes linked to glutamate exocytosis. Here we investigate whether the transcriptional profile of this specific astrocyte subtype is altered across various AD mouse models.

**Method:**

We conducted differential gene expression analysis and cellular deconvolution using population‐specific expression analysis (PSEA) on bulk RNA‐seq data from the hippocampus of three amyloidosis models: APP/PS1 (*n* = 18), 5xFAD (*n* = 49), and hAβKi (*n* = 41). Cell type‐specific gene expression signatures for astrocytes, neurons, oligodendrocytes, endothelial cells, and microglia were assigned using the BRETIGEA R package. The transcriptional signature for glutamatergic gliotransmission‐specialized astrocytes consisted of 15 genes associated with synaptic‐like glutamate release machinery. We analyzed cellular enrichment proportions and identified differentially expressed genes (DEGs) within the glutamatergic astrocyte signature across the three models (*p* < 0.05).

**Result:**

The APP/PS1 model showed the highest glutamatergic astrocyte enrichment (24.8%), similar to hAβKI (23.8%), while 5xFAD had minimal enrichment (2.2%) (Figure 1). From these astrocytic genes, DEGs analysis revealed 52 upregulated and 547 downregulated genes in the APP/PS1 model, 24 upregulated and 36 downregulated genes in the hAβKI model, and 10 upregulated and 8 downregulated genes in the 5xFAD model. Notably, genes like *KIF5B* and *GRASP* were significantly downregulated, indicating impairment in vesicle homeostasis. The most significantly up‐ or downregulated genes differed across the three models (Figure 2).

**Conclusion:**

Our results show that the glutamatergic astrocyte signature varies in AD mouse models, with significant differences in their enrichment and gene expression profiles. The APP/PS1 and hAβKI models showed substantial enrichment of this astrocyte subtype, whereas the 5xFAD model exhibited minimal representation. The significant downregulation of genes related to vesicle trafficking suggests potential impairments in glutamatergic astrocyte function. Our findings highlight the role of a specific astrocyte subtype in AD pathogenesis. Further single‐cell analysis will provide more information at the cellular level.